# Correction to “Differential activity of MEK and ERK inhibitors in BRAF inhibitor resistant melanoma”

**DOI:** 10.1002/1878-0261.70297

**Published:** 2026-07-12

Carlino MS , Todd JR , Gowrishankar K , Mijatov B , Pupo GM , Fung C , et al. Differential activity of MEK and ERK inhibitors in BRAF inhibitor resistant melanoma. Mol Oncol. 2014;8(3):544–54. 10.1016/j.molonc.2014.01.003.24476679 PMC5528644

In the article, an error occurred during figure assembly that resulted in the Western blot images in Figure 2C being published with a duplicated *β*‐actin control.

The authors have provided a technical replicate for the Western blot experiment along with a corrected version of Figure 2C, as shown below. The revised data are consistent with the findings reported in the original manuscript and confirm that no PARP cleavage is observed in the RTK‐activated melanoma cells (M238R1 and CR201) in response to MEK or ERK inhibition.

The legends for Figures 2B and 4A have also been updated to clarify that target proteins were resolved across separate blots using identical lysates. As a result, multiple *β*‐actin loading controls may appear for the same samples, reflecting the individual gels used for protein analysis.

The authors agree to this corrigendum and confirm that these changes do not affect the conclusions of the article. The authors apologize for any inconvenience caused.

The corrected version of Figure 2C and updated captions for Figures 2 and 4 are provided below:

**Fig. 2C:**

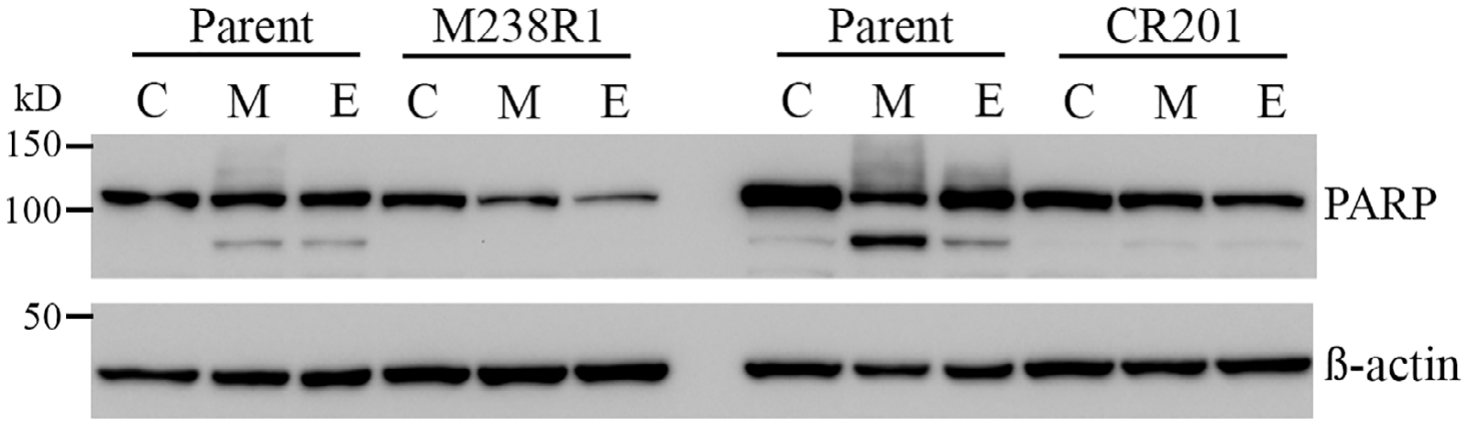

**Fig. 2.** Resistant melanomas with RTK activation retain anti‐proliferative sensitivity to ERK but not MEK inhibition with no apoptotic sensitivity to either inhibitor A. Viability curves of the parental and isogenic melanoma sublines treated with indicated concentrations of the MEK inhibitor trametinib and ERK inhibitor VX‐11e for 72 h (relative to DMSO‐treated controls; mean ± SD; *n* = 2). B. Parental and BRAF inhibitor‐resistant sublines were treated with DMSO (C), 10 nm MEK inhibitor trametinib (M) or 10 mm ERK inhibitor VX‐11e (E) for 24 h. Western blots of lysates showing protein markers of MAPK activity and cell cycle progression. C. PARP cleavage was determined 72 h after treating sublines with inhibitors as described above. Both full length and major cleaved PARP proteins shown. D. Cell cycle distribution of the indicated cell lines treated with either DMSO (control), 10 nm trametinib (MEK inhibitor), or 10 mm VX‐11e (ERK inhibitor) for 72 h (mean ± SD; *n* = 4). For Western blot analysis, target proteins were resolved across separate blots using identical lysates, and a representative ß‐actin loading control is shown. *Significant differences for subG1 and S phase when MEK compared to ERK inhibitor treatment (*P* < 0.05).

**Fig. 4.** Combination ERK inhibitor with BEZ235 is more active than the MEK inhibitor/BEZ235 combination at promoting cell death A. The indicated cell lines were treated DMSO (C), 10 nm trametinib (M), or 10 mm VX‐11e (E) for 24 h. Western blots of lysates showing protein markers of MAPK and AKT activity. WMD013 and Patient 3 cell lines were directly compared to SKMel28 and BR2 cell lines. B. Whole cell lysates from the indicated cell lines were subjected to pull‐down (PD) assays with GST‐bound CRAF RAS‐binding domain after 24‐h treatment with DMSO (C) or 10 mm VX‐11e (E). Whole cell lysates (total RAS) and pull‐down products (activated RAS) were immunoblotted with a pan‐RAS antibody. C. Histogram showing the percentage sub‐G1 population of cell lines treated with either DMSO (control), 10 nm trametinib (MEK inhibitor), 10 mm VX‐11e (ERK inhibitor), 2 um BEZ235 or combinations as indicated for 72 h (mean ± SD; *n* = 4). For Western blot analysis, target proteins were resolved across separate blots using identical lysates, and a representative ß‐actin loading control is shown. *Significant differences between the ERK/BEZ235 inhibitor combination compared with the MEK/BEZ235 inhibitor combination (*P* < 0.05).

